# Using Bluetooth proximity sensing to determine where office workers spend time at work

**DOI:** 10.1371/journal.pone.0193971

**Published:** 2018-03-07

**Authors:** Bronwyn K. Clark, Elisabeth A. Winkler, Charlotte L. Brakenridge, Stewart G. Trost, Genevieve N. Healy

**Affiliations:** 1 The University of Queensland, School of Public Health, Herston, Queensland, Australia; 2 Institute of Health and Biomedical Innovation at Queensland, Centre for Children’s Health Research, Queensland University of Technology, South Brisbane, Queensland, Australia; Indiana University, UNITED STATES

## Abstract

**Background:**

Most wearable devices that measure movement in workplaces cannot determine the context in which people spend time. This study examined the accuracy of Bluetooth sensing (10-second intervals) via the ActiGraph GT9X Link monitor to determine location in an office setting, using two simple, bespoke algorithms.

**Methods:**

For one work day (mean±SD 6.2±1.1 hours), 30 office workers (30% men, aged 38±11 years) simultaneously wore chest-mounted cameras (video recording) and Bluetooth-enabled monitors (initialised as receivers) on the wrist and thigh. Additional monitors (initialised as beacons) were placed in the entry, kitchen, photocopy room, corridors, and the wearer’s office. Firstly, participant presence/absence at each location was predicted from the presence/absence of signals at that location (ignoring all other signals). Secondly, using the information gathered at multiple locations simultaneously, a simple heuristic model was used to predict at which location the participant was present. The Bluetooth-determined location for each algorithm was tested against the camera in terms of F-scores.

**Results:**

When considering locations individually, the accuracy obtained was excellent in the office (F-score = 0.98 and 0.97 for thigh and wrist positions) but poor in other locations (F-score = 0.04 to 0.36), stemming primarily from a high false positive rate. The multi-location algorithm exhibited high accuracy for the office location (F-score = 0.97 for both wear positions). It also improved the F-scores obtained in the remaining locations, but not always to levels indicating good accuracy (e.g., F-score for photocopy room ≈0.1 in both wear positions).

**Conclusions:**

The Bluetooth signalling function shows promise for determining where workers spend most of their time (i.e., their office). Placing beacons in multiple locations and using a rule-based decision model improved classification accuracy; however, for workplace locations visited infrequently or with considerable movement, accuracy was below desirable levels. Further development of algorithms is warranted.

## Introduction

How time is distributed between sedentary and physically active behaviours impacts on our health and wellbeing. Too much sitting is detrimentally linked with cardiovascular health, mental health, diabetes mellitus, obesity, cancer and premature mortality [1, 2]. Conversely, being active, even at light intensities such as standing [3, 4], can be of benefit. Objective activity monitors, which have been increasingly used in population based studies [5], have provided important insights into the duration and time of day spent in these various activities, [6]. However, although these monitors provide a significant advance over self-report measures in terms of their accuracy [7], most do not capture data on the contexts of behaviours, that is, where the behaviour is occurring. Measuring movement within specific physical contexts may help to develop more effective behaviour change strategies by enabling interventions to target the locations in which prolonged sitting is most likely to occur, and/or the locations in which participants most frequently make behaviour changes. For example, if most sitting time occurs in the office, instituting changes such as sit-stand desks could be used. Alternatively, if high sitting time occurs in the kitchen/lunch room, then providing standing tables and/or changing practices for lunch time (e.g. eating outside; doing activity during the lunch break) could be an effective strategy to enhance movement.

At present, assessment of time spent in various locations is often self-reported and may be subject to error. Global positioning systems can, to some extent, measure a person’s location, and, thereby, the physical context in which physical activity and sitting time occurs [8]. However, this technology is not accurate within buildings where the signal may be lost [8]. Other methods better suited to indoor measurements have been trialled, such as wireless location systems (e.g., radio frequency identification tags; RFID) [9] and wearable cameras [10]. However, authors of papers on both of these approaches have reported difficulties in obtaining usable data [9, 11], with linking to activity data proving difficult with RFID tags and coding the large amounts of information generated from cameras remaining thus far difficult to automate.

One other technology that has been identified with potential to determine indoor location is Bluetooth signalling [8]. Bluetooth signals can be used to detect whether or not beacon and receiver device are in proximity of each another; however, the manner in which they operate means their proximity detection does not correspond to consistent, fixed spatial distances [12]. Consequently, they have the potential to measure the physical setting in which the wearer’s activity and sitting time occurs [8], but with an unknown degree of accuracy. Recently, Bluetooth has been added as a function of activity monitors, for example in the ActiGraph GT3X + BT and GT9X Link (www.ActiGraphcorp.com/) monitors. Having both activity monitoring and proximity location functions in one device is potentially advantageous to researchers (ease of use and data already linked), and participants (lower burden). Such devices have been employed to provide summaries of activity occurring within various locations, including within the office [13] and within a nursing home environment [13]. However, to our knowledge, the accuracy of proximity data derived from Bluetooth-enabled accelerometers to determine location is not yet established in the published literature.

This current study aimed to address this limitation by examining the accuracy of Bluetooth sensing to determine the physical context of behaviour in a workplace setting using the proximity-detection function of the ActiGraph GT9X Link accelerometer. The workplace was chosen as it has been identified as a key setting in which to address prolonged sitting time [14], and the intervention strategies to achieve reductions in sitting may be applied in multiple locations within the work space (e.g. at the desk, in meeting rooms, in the lunch room). The accuracy of two data collection approaches was evaluated, both chosen in consideration of the practicalities of applying this method within the free-living context. The first approach was to use signals from a beacon or beacons in a single location (termed one-location-monitored throughout the paper). Such an approach may be appropriate when a single priority location needs to be assessed, such as an entryway to a floor or building (in an attempt to capture when participants enter and leave) or the participant’s office (to assess time spent sitting in the office). The second approach, termed multiple-locations-monitored, used all of the information from signals from beacons placed in multiple locations across the workplace—specifically the locations that were commonly occupied in that particular workplace—and then predicted at which of these locations the participant was present. Such an approach might be implemented when it is of interest to measure the time spent in multiple neighbouring locations, or the activity occurring in these locations. A secondary aim was to assess the accuracy of both approaches when monitors were worn as receivers on the wrist (a wear position often chosen to improve wear compliance [15]) or on the thigh (a wear position with the potential to accurately measure sitting time and postural transitions [16]).

## Materials and methods

### Participants

**Fig 1:**
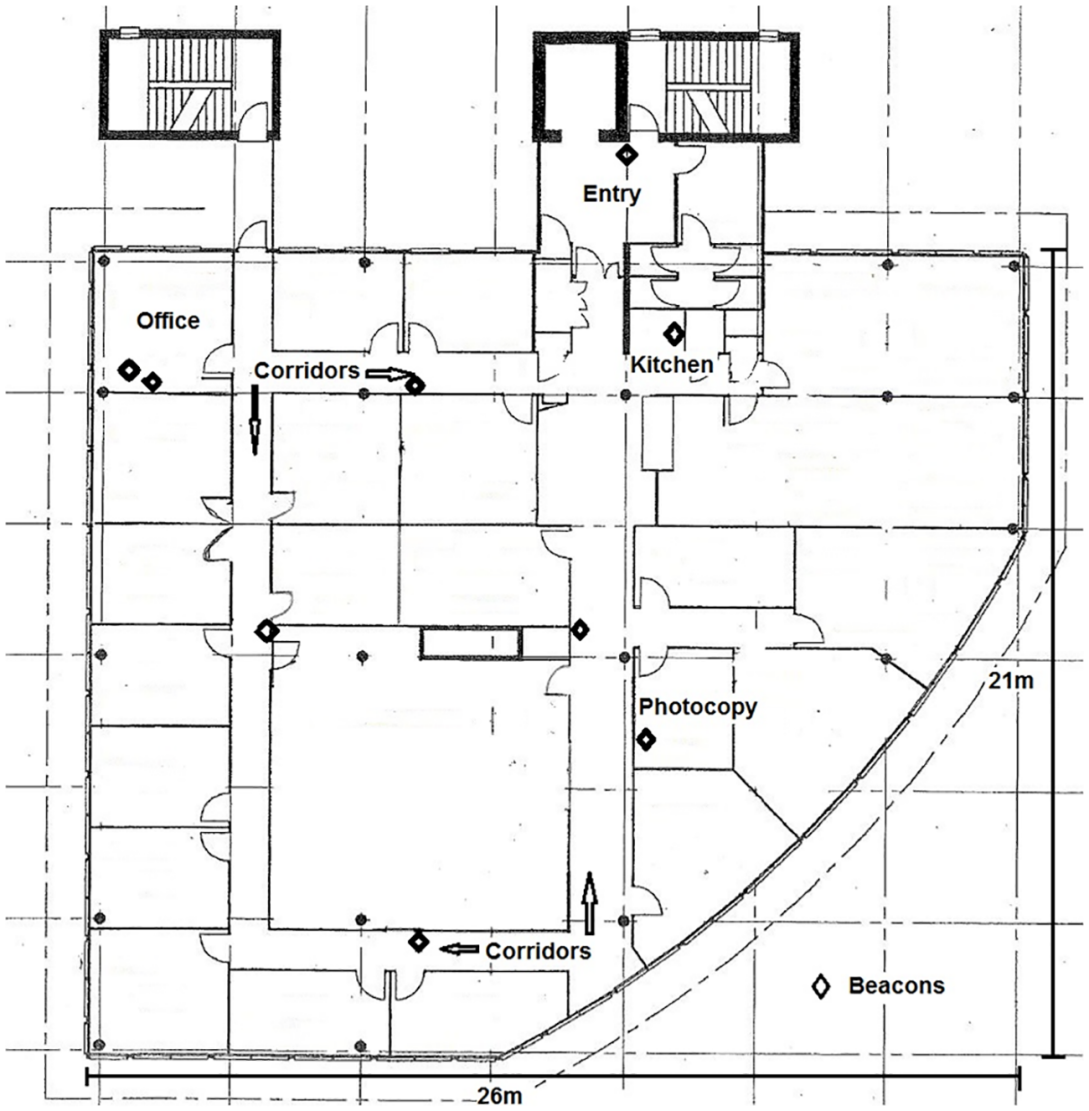
Typical layout of workplace floor and beacon placement.

Thirty participants were recruited into the *Where and When at Work* study from University staff (from one workplace building) by convenience sampling methods including word of mouth, posters and email. Those who showed interest received an information sheet that explained the study, including its eligibility criteria, attached to an email invitation to join the study. To be eligible for the study, participants had to be aged over 18 years, ambulatory and work at least one day in the test building. Eligible participants (eligibility confirmed by the research team) provided written informed consent prior to enrolling in the study. Ethical clearance was obtained from Bellberry Limited (Protocol 2016-01-009). Participants were recruited from all five floors of the building, each of which had slightly different layouts. A typical layout is shown in Fig 1.

### Data collection

Participants were contacted to schedule the day of data collection and were asked to identify the places in which they commonly worked. The data collection protocol involved a single day of monitoring during work hours. Participants were asked to visit the research project office prior to starting work on the day of monitoring where they were given a questionnaire and fitted with two ActiGraph GT9X Link activity monitors, an activPAL3 micro device and a wearable camera. Participants were instructed to perform their usual work tasks and to return to the research office at the end of the work day, when the questionnaire, camera and monitors were collected. Monitors had been initialised to record time in a consistent manner, using the same computer, but thereafter recorded time independently. Data collection was carried out from August to October 2016.

### Measures

#### Questionnaire

Age, gender, education, employment status, height, and weight was captured via the self-completed questionnaire. Body mass index (BMI) was determined as weight (kg)/height (m)^2^.

#### Wearable camera

Each participant wore the MeCam camera, which weighs only 50g, attached to the chest with a clip. This camera captures the environment of the wearer, recording videos in MP4 file format and storing them in an external memory card of up to 64 GB (http://mecam.me/). The camera is controlled via an iOS or Android platform through the MeCam HD application and via on/off buttons on the camera. As recording drains the battery quickly, researchers visited the participants at 60–90 minute intervals to replace the used camera with a new one, thereby achieving continuous recording of the whole day at work. Due to ethical issues, video recording was not allowed outside the test building and, therefore, meetings or work that took place outside the building were not recorded. Additionally, participants were asked to turn off the cameras when involved in potentially sensitive work and for bathroom visits. Researchers recorded the camera replacement times and participants were asked to report if the camera had been turned off or they had left the building. Each period of recording from the cameras was transferred to a research computer and files were stored securely on a shared drive.

#### ActiGraph GT9X Link monitor

The ActiGraph GT9X Link monitor is a small lightweight (14g; 3.5 x 3.5 x 1 cm) accelerometer with Bluetooth enabled functions (www.ActiGraphcorp.com/). One such function is the detection of proximity between two devices. Monitors can be initialised as receivers, beacons or both. Monitors initialised as beacons transmit the signal with their serial number to those initialised as receivers at predetermined intervals (e.g., 10s or 1min). Receivers then store time-stamped received signal strength values (Received Signal Strength Indicator; RSSI) that have been detected from nearby beacons identified by beacon serial numbers. RSSI is a term used to measure the relative quality of a received signal (in this case Bluetooth) to a device but does not have an absolute value. ActiGraph states that ‘RSSI value is not directly proportional to Bluetooth signal strength’ [17]. All monitors (beacons and receivers) were initialised on the ActiGraph software platform, ActiLife 6 (version 6.13.2).

Monitors initialised as receivers: Participants wore two monitors initialised as receivers to collect beacon signals at 10-second intervals (the shortest interval available on ActiLife at initialisation). One receiver was worn on the left wrist on a wrist band. The other receiver was taped to the right thigh (front medial aspect, approximately one third of the way from hip flexor crease to knee) with Hypafix^™^ adhesive tape. Data from the receivers were downloaded and exported as comma separated value files which contained, for each 10-second interval, time-stamped recordings of movement (triaxial acceleration) and proximity (RSSI strength for each beacon signal; see example of plotted RSSI output values for each location in one hour for a single participant in S1 Fig).

Monitors initialised as beacons: Beacons were placed in locations that would likely capture most of this sample’s work time based on participants’ responses to where they commonly spent time at work: the corridors; the kitchen area; the photocopy room; and, the office. Only the locations on the participants’ own floor were monitored and “office” refers to the participant’s own office (not the office of another worker). A single beacon was placed in the kitchen, photocopy room and in each segment of the corridors (with two to four corridor beacons used in total, depending on the floor layout). Two beacons were placed in each participant’s office—one on the desk and one on a nearby wall—in order to explore the optimal beacon placement (desk, wall, or both) in this key location in which participants were likely to spend much of their time. All beacons were attached with Blu-tack^™^ adhesive to the walls at a height of approximately 150cm, or to the front aspect of the office desk. The number of floors and participants assessed simultaneously varied from one participant on one floor to three participants across two floors. The building configuration is such that the commonly used spaces for each floor all lie together in the main portion of the floor (350–550 square metres), which can only be entered only via a single entryway area on each floor (12 square metres) as per Fig 1. A single beacon was also placed in the floor entryway area, with the goal of determining whether the accuracy in this location was sufficient to estimate when participants are at work from entry and exit patterns measured in this manner.

#### activPAL3 device

Trained staff affixed the activPAL3 micro to the participant’s thigh using hypoallergenic medical dressing. The device was placed at the recommended position of one third of the way down the thigh along the midline, underneath and on the same leg as the thigh-worn receiver monitor. The activPAL was initialised and downloaded in PAL version 7.2.32 (PAL Technologies Ltd., Glasgow, Scotland, UK). Due to device failure one participant did not have activPAL3 data available.

### Data processing

#### Wearable camera

Project staff familiar with the workplace and its spaces viewed the video recordings and classified the location of the participant. The classifications were obtained for every 10-second observation, as per the timestamp from the receiver monitors’ proximity file (downloaded from ActiLife). The classifications were applied to the entire 10-second observation when calculating time spent in each location. Locations were coded as: entry, office; kitchen; photocopy room; corridor; on office floor but not in the aforementioned locations; outside office floor; no camera recording; and, unable to be determined. For comparison with the monitor-determined positions, unknown locations were excluded (i.e., images that were unable to be coded or no camera recording). Locations determined by the wearable camera were used as the ground truth for location.

#### ActiGraph Link monitors

The accuracy of proximity detection via the monitors, relative to location as determined by wearable camera, was assessed for two different beacon configurations: the one-location-monitored approach, and the multiple-locations-monitored approach.

For the one-location-monitored approach, signals received from beacons in locations other than the one of interest were ignored. The participant’s presence or absence at a given location was predicted from whether or not a signal was detected from any beacon in that location. Signal presence or absence was used rather than using the continuous RSSI values, consistent with the lack of a consistent, predictable relationship between RSSI values and degree of physical proximity [12]. Accuracy for the office location was evaluated under three possible conditions: wall bacon only; desk beacon only; and, desk and wall beacon.

The multiple-locations-monitored approach used the information from the multiple beacons in the work space to predict participant location. While a participant can only be in one location at a time, signals can be detected in multiple locations simultaneously. For this situation, a simple heuristic model was used so that only a single participant location per 10-second epoch would be selected. If, during a particular epoch, signals were detected in only one location, the model selected the signal location as the participant location. Alternatively, if signals were not present in any of the locations, the participant location was assigned a code of “workplace-other”. If signals were detected in multiple locations simultaneously during a particular epoch, the participant location was selected using a majority vote as the location at which a signal was most frequently detected over a moving time window before and after the epoch in question (t). Several time windows, covering a range of possible total durations between 10 seconds and 4 minutes were trialled: t±0 to t±5, t±9 and t±12 10-second epochs. If multiple locations met the criteria for “most frequent” over the time window, participant location was assigned by selecting among these by applying the following preferential order: office; corridors; kitchen; then, photocopy room. The order was chosen by ranking locations by their prevalence of signal presence across the entire sample, with this ranking serving as a simple indicator of the underlying relative likelihood that a participant is in each location.

#### activPAL3 device

The activPAL3 data from the events files were extracted over the timeframe captured by the video camera, wrist- and thigh-worn receivers, and summarised by camera-determined location (using the same 10-second observations as per the proximity files for the receiver monitors). For each location, total time spent sitting and standing were determined, along with time spent stepping, which was reported separately as purposeful walking when it occurred continuously for at least 30 seconds [18] or otherwise as incidental stepping. This data was used to describe the posture and movement of participants in each location assessed.

### Statistical analyses

Data processing and analyses were performed in SAS 9.4 (SAS Institute Inc, Cary, NC, US). The SAS code used to implement the multiple-locations-monitored decision model is presented in Supplemental Material (S1 File). Sample descriptives including participant characteristics, posture, movement and location usage were reported as n (%) and mean and standard deviation (SD). Additionally the w50%, a measure of the bout duration where half of all time at each location is accumulated through being there this amount of time or longer [19], was used to describe how long at a time participants spent in each location.

The locations determined from signals received by the thigh- and wrist-worn monitors were compared with the direct observation criterion for agreement, limited to the times that location could be determined by the video recording criterion measure (n = 67616 observations, approximately 188 hours in total). Recall, also known as sensitivity, quantified how commonly the monitors correctly detected the participant’s presence when participants were in a particular location. This was calculated as true positives/(true positives + false negatives). Precision, also known as positive predictive value, quantified how commonly a participant was genuinely present when the monitor predicted the participant was at a particular location. This was calculated as true positives/(true positives + false positives). Overall performance was determined by the F-score calculated as 2 x ((recall x precision)/(recall+precision))[20]. The proportion of classifications that were correct ((true positives + true negatives)/all observations) was also reported.

For the multiple-locations monitored approach, performance was evaluated using a variation of k-fold cross-validation [21]. The dataset was randomly partitioned, by subject, into five folds (n = 6). Four-fifths of the data were used to identify the window size that generated the highest F-scores across locations, with the remaining one-fifth used for performance evaluation. The procedure was repeated 5 times so that all 5-folds were used once as a tuning and testing dataset. The overall performance accuracy was based on the mean (±SD) agreement statistics from each test fold (N = 30). The analyses treated the times recorded by each device as though they are synchronised. Supporting this approach, we saw that time-shifting each camera’s data by ±30 seconds to whatever maximised device-camera agreement for office time frequently did not improve F scores in general and also frequently identified zero seconds as the optimal time shift (S1 Table).

## Results

Most participants were female (n = 21, 70%) and worked full time (n = 23, 77%), and nearly all had a post-school qualification (n = 29, 97%). Participants had a mean (±standard deviation) age of 38.3 (±11.3) years, BMI of 23.3 (±4.1) kg/m^2^ and were monitored by video camera for on average 6.3 (±1.2) hours which was 88% of work hours (7.2 (±1.1) hours). The primary reasons for not recording all work hours were time spent outside the building (not allowed to record due to privacy considerations) and battery failure on the video camera.

Table 1 shows the attributes, usage and movement with five common workplace locations. Average daily time use (measured by video) in each of the commonly reported areas ranged from only 2.1±4.5 minutes (1%) in the photocopy room to 319.2±86.3 minutes (93%) in the participant’s office. On average, participants spent very little time in the entryway (1.5±0.8 min). Participants tended to accrue time in their own office by being there continuously for a long time (w50% = 49.8 minutes) with time nearly entirely spent sitting (80%) or standing (18%). By contrast, their visits to the other locations were much shorter, with all the other w50% under five minutes (Table 1). Presence in a particular location was extremely brief (i.e., for only one 10-second period at a time) for only 0.4% of observations in the office, for 5–10% of observations in the other commonly used areas, and for 76.5% of observations in the entryway. Standing was the most common activity in the corridors, kitchen and photocopy room, while purposeful walking was the most common activity in the entryway, and sitting accounted for most of the time spent in the office (Table 1).

**Table 1:** Attributes, usage and movement within five common workplace locations (n = 30 participants).

	Office	Corridors	Kitchen	Photocopy	Entry
**Attributes**					
Identified as common?^a^	Yes	Yes	Yes	Yes	No
Multiple entries and exits?	No	Yes	Yes	No	Yes
Contains seating?	Yes	Yes	Some floors	No	Some floors
**Usage (video camera)**^b^					
Participants who visited, *n*	30	30	26	18	30
Mean ±SD time occupied, *min*	319.2 ±86.3	15.5±16.9	5.6±7.4	2.1±4.5	1.5±0.8
w50% ^c^, *min*	49.8	4.9	3.2	1.1	<0.1
**Movement (activPAL3)**^a^					
% of time in location spent…					
Sitting	80%	26%	18%	3%	8%
Standing	18%	49%	69%	72%	27%
Incidental stepping ^d^	1%	13%	10%	19%	16%
Purposeful walking ^d^	1%	11%	3%	7%	49%

^a^Participants identified as commonly used space.

^**b**^All time within each 10-second time interval was assigned to the location measured during that ten second interval

^**c**^ 50% of time at the location is accumulated by being there for a period of w50% or longer.

^d^ Walking for <30 or ≥30 seconds at a time are respectively identified as incidental stepping and purposeful walking

### One-location-monitored approach

When using the one-location-monitored approach (Table 2), classification accuracy varied widely by location and differed somewhat depending on whether the receiver was worn on the thigh or the wrist. The wrist-worn receiver tended to have a slightly lower classification accuracy than the thigh-worn receiver overall, due to lower precision, but not at every location, and it sometimes showed greater recall than the thigh-worn receiver. Consistently, the most accurate classification obtained was for the office location and the worst classification was obtained for the photocopy room. Placing a single beacon in the entryway area was not an accurate method to detect a participant’s presence in this area, with overall F-scores of only 0.24 (thigh) and 0.14 (wrist). For each location with low performance, the main issue was very low precision, with a large number of false positives (Table 2). A high false negative rate (evidenced by the low recall) was additionally an issue in the locations that largely serve as a transition to other spaces and where participants spent most time moving (the entryway and the corridors).

**Table 2:** Agreement with direct observation of the monitor-ascertained presence/absence at each location, using beacons from one location at a time (n = 30 participants, n = 67616 observations).

	Location	Observations (Camera)	Correct^a^	Recall^b^	Precision^c^	F-score^d^
Thigh monitor	Office	57454	0.958	0.984	0.967	0.976
	Kitchen	1013	0.953	0.877	0.225	0.358
	Corridors	2791	0.775	0.427	0.081	0.136
	Photocopy room	385	0.890	0.894	0.044	0.085
	Entry	277	0.991	0.361	0.181	0.242
Wrist monitor	Office	57454	0.949	0.991	0.951	0.970
	Kitchen	1013	0.856	0.946	0.090	0.165
	Corridors	2792	0.519	0.732	0.060	0.112
	Photocopy room	385	0.752	0.956	0.021	0.042
	Entry	277	0.974	0.509	0.081	0.140

^a^ (true positives + true negatives)/(true positives + false positives + true negatives + false negatives)

^b^ true positives/(true positives + false negatives)

^c^ true positives/(true positives + false positives)

^d^ 2x(recall x precision)/(recall + precision)

When wearing the receiver on the thigh, the use of a single beacon mounted either on the wall or desk achieved F-scores of 0.95, slightly lower than when using both beacons (F = 0.98). With wrist-worn receivers, all options resulted in F scores of approximately 0.96–0.97 with the F score highest by a small margin when using both beacons (F = 0.97).

### Multiple-locations-monitored approach

**Fig 2:**
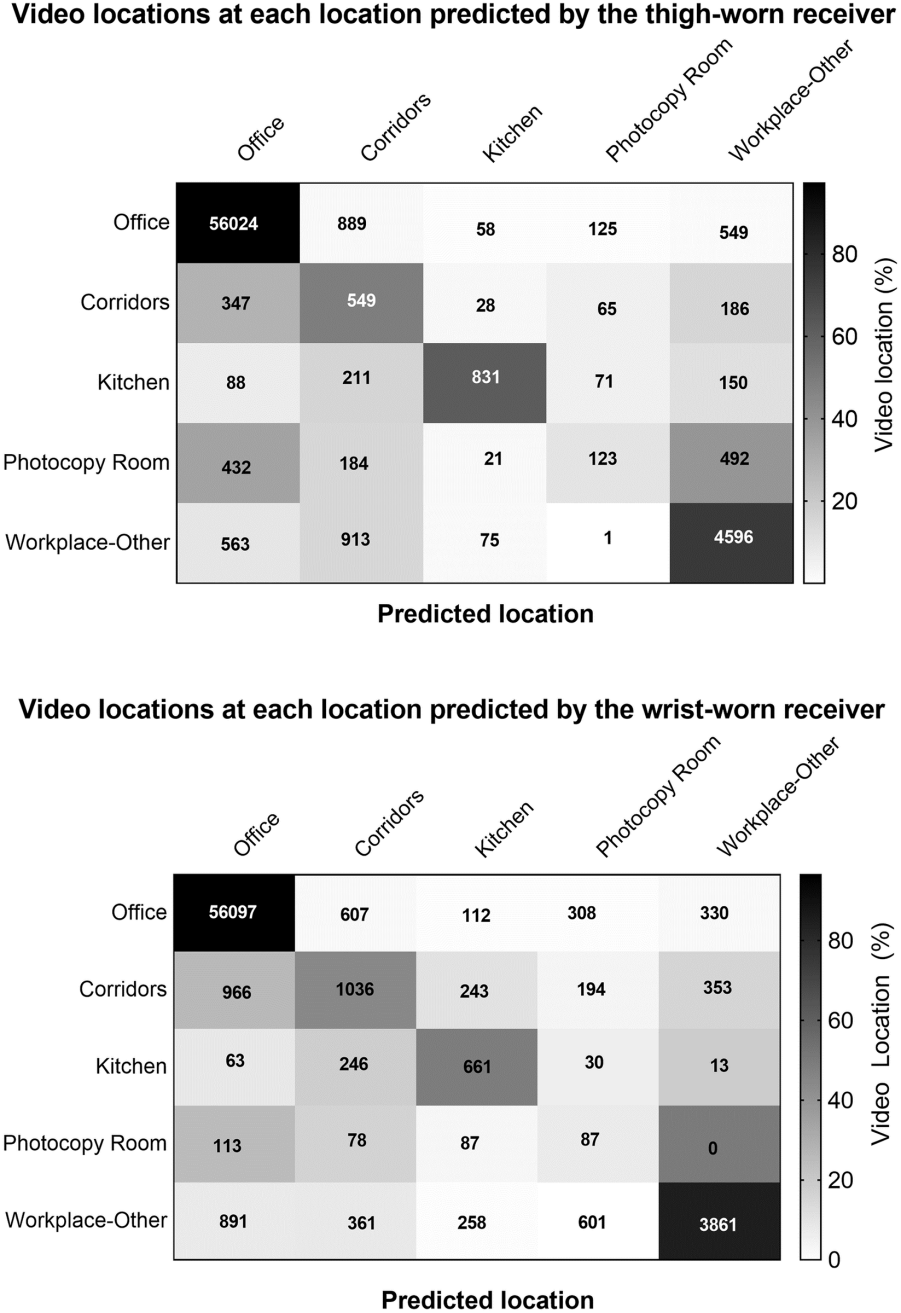
Location predicted by heuristic model using beacon signals received by the thigh and wrist-worn monitors compared with actual location determined by video camera recordings.

The heuristic model chosen used a window of t±4 10-second epochs (S2 Fig). A model to select between multiple locations was necessary as signals were simultaneously present in multiple locations for much of the time (61%, n = 41380 observations and 31%, n = 20785 for the wrist- and thigh-worn receivers, respectively). In the chosen model, ties occurred for 35% and 10% of all classifications for wrist and thigh worn receivers respectively, therefore requiring the decision rule based on prevalence to select among tied locations for these epochs. For receivers worn in both positions, the heuristic model provided near identical F-scores for the office and higher F scores for all the other locations (Table 3) relative to the one-location-monitored approach. The greatest F-score improvements was seen in the kitchen and corridor classifications (improvement 0.15–0.31) with a small improvement in the photocopy room (0.07–0.08). The best performance was observed for “office” (F-score = 0.97–0.97), followed by “workplace-other” (F-score = 0.64–0.70) with lower performance accuracy in the other locations, especially the photocopy room (F-score = 0.11–0.17). A figure displaying the predicted location by actual location is shown in Fig 2. The multiple-locations monitored approach was able to identify the location of the participants 91% of the time using the wrist worn receiver and 92% of the time using the thigh worn receiver.

**Table 3:** Agreement between the monitor determined location and direct observation when measuring multiple locations, using the decision model (Mean±SD across the 5 holdout samples of n = 6 participants).

	Location	Correct^a^	Recall ^b^	Precision^c^	F-score^d^	Difference^e^
Thigh monitor	Office	0.955 ± 0.016	0.975 ± 0.015	0.972 ± 0.013	0.973 ± 0.010	-0.003
	Kitchen	0.99 ± 0.005	0.818 ± 0.049	0.570 ± 0.097	0.668 ± 0.072	0.310
	Corridors	0.958 ± 0.016	0.218 ± 0.064	0.469 ± 0.146	0.289 ± 0.067	0.153
	Photocopy room	0.98 ± 0.016	0.278 ± 0.169	0.156 ± 0.096	0.165 ± 0.104	0.080
	Workplace-other	0.956 ± 0.021	0.703 ± 0.217	0.702 ± 0.231	0.699 ± 0.220	n/a
Wrist monitor	Office	0.950 ± 0.010	0.977 ± 0.008	0.965 ± 0.015	0.971 ± 0.005	0.001
	Kitchen	0.985 ± 0.006	0.572 ± 0.252	0.401 ± 0.182	0.469 ± 0.207	0.304
	Corridors	0.955 ± 0.006	0.358 ± 0.097	0.433 ± 0.227	0.373 ± 0.126	0.261
	Photocopy room	0.979 ± 0.016	0.187 ± 0.137	0.094 ± 0.072	0.109 ± 0.074	0.067
	Workplace-other	0.958 ± 0.019	0.567 ± 0.284	0.785 ± 0.208	0.642 ± 0.246	n/a

^a^ (true positives + true negatives)/(true positives + false positives + true negatives + false negatives)

^b^ true positives/(true positives + false negatives)

^c^ true positives/(true positives + false positives)

^d^ 2x(recall x precision)/(recall + precision)

^e^ Improvement from corresponding F-scores in Table 2 for examining a single location only.

## Discussion

To our knowledge this study is among the first to test the accuracy of Bluetooth sensing using the proximity function of the ActiGraph GT9X Link for measuring location within an office-based workplace. The accuracy, using video observation as a criterion, depended on the amount of time participants spent in each location, whether signals from beacons in multiple locations or from one only location were used to predict location, and to a lesser degree, whether the monitor was worn as a receiver on the thigh (better) or wrist. Overall, when employing the multiple-locations-monitored approach, Bluetooth sensing was able to correctly identify the location of office-based workers out of five potential locations over 90% of the time for both receiver wear positions. However, some locations were predicted much more accurately than others. The location best detected was the office in which participants were most stationary (sitting and standing) and present continuously for the longest time (w50% = 49.8 min), while the other locations that were detected more poorly were those in which participants moved more and passed through more quickly (w50% <0.1 min to 4.9 min). These findings will help inform measures of sitting and standing by providing contextual information in addition to duration, which can already be measured by monitor.

Much of the participants’ time was spent in their own offices (93%). It appears to be highly feasible to measure time spent in the office with acceptable accuracy, by placing one or two beacons in this location, with or without placing beacons at other locations around the workplace, when using either wrist- or thigh-worn receivers. As such, it is likely that measures of sedentary behaviour and activity in the office can be obtained with a similar degree of accuracy to overall measures of sedentary behaviour and activity. None of the options that were evaluated were suitable for measuring the time participants spent in the other locations, and consequently movements within these locations. Using the heuristic algorithm to determine a single location from multiple Bluetooth signals improved accuracy, but not to levels that provided accurate classifications for all locations.

Accuracy may have been highest for the office because participants were frequently there (limiting the opportunity for false negatives), because participants spent long periods continuously there (limiting problems arising from Bluetooth signals being intermittent) and/or because participants were mostly stationary while in the office (either sitting or standing). Movement has been shown to effect the reliability of RSSI signals with less reliable signals received at greater walk speeds (maximum velocity 2m/s) than stationary or slow walk speeds (maximum velocity 0.22m/s) [22]. Future research should examine the accuracy of location detection in workers that may spend moderate periods of time in different activities and in multiple locations, for example in Activity Based Working office designs.

Placing a single beacon in an entryway showed little promise as a method for inferring time at work from when participants arrived at and left their workplace (taking their floor as their workplace). Since the time spent continuously in the entryway was very short, unsurprisingly, the proximity data collected in 10-second intervals often failed to detect that participants were in this space (i.e., recall was low). By contrast, when signals were completely absent for at least one epoch (10 seconds) from the office, corridors, photocopy room and kitchen, this indicated with reasonable accuracy (F≈0.6–0.7) that the participants were not in any of these areas (i.e., “workplace-other”). It is possible that worker presence/absence from the workplace might be captured reasonably well by signal presence/absence for a sustained period of time across a comprehensive set of beacons, placed in appropriate locations.

Applying a simple heuristic algorithm to the signals collected from beacons in multiple locations was able to improve classification accuracy somewhat. Algorithm development might further improve accuracy. One potential area for improvement that is highly feasible may be to improve the tie-breaking rule. This rule had informed nearly 40% of classifications from the wrist-worn receiver, but was based on a very simple ranking of overall group-level prevalence. Simple ideas such as individualising the prevalence rankings could be trialled, as could more complex rules, such as considering the specific combinations of locations detected and/or their temporal sequence. The current method did not take in to account movement data from the accelerometer and including such information may improve methods to determine location. Indeed the future of assessing context for public health research may lie in utilising data from multiple sensing technologies such as has been suggested in a review of technology to identify activities of daily living [23].

Future method development may also take into account actual RSSI values rather than reducing them to present/absent. There is no simple correspondence between continuous RSSI signals and the physical distance between the beacon and receiver, due to surfaces and physical barriers in the environment [12] and sources of variation in measures taken within a single location [24]. However, recent literature has shown that the accuracy of location prediction can improve when using RSSI signal strength in localisation algorithms [25]. Such methods include multilateration—which measures signal strength received from several different beacons—and fingerprinting—which involves pre-recording RSSI values from several points with in the location and using this data to create a characteristic feature for each location. When used to classify the room location of a stationary piece of equipment in a hospital setting fingerprinting provided higher percent correct location classification (79%) than multilateration (61%) [25]. When it is possible to undertake a detailed examination of how RSSI values vary within the physical environment in question prior to data collection, fingerprinting may provide improvements in location accuracy.

There is further potential for the proximity function of the ActiGraph GT9X Link monitors to be set up to detect interactions between people when initialised as both beacon and receiver. Using Bluetooth signalling for detecting interaction has been demonstrated in a study using the Sociometer to detect proximity between staff in simulated interactions in a hospital setting with moderate accuracy (percent interactions correctly identified: 77%; range 55–95%) [26]. The use of a single device to measure both movement and the physical context of the behaviour, as well as potentially capturing interactions between people, provides benefit not only in terms of the wealth of detailed data collected, but also in relation to lowering the burden to researchers and participants. Such information could be used for assessing the impact of interventions to increase movement and decrease sitting time by showing where changes are made and how peer support influences those changes. This information would also be useful in assessing innovative building design by helping designers understand the flow and interactions between workers when spaces are designed to be conducive to activity based and interactive work practices.

The main strength of this study is the assessment of accuracy under free-living conditions. The use of a wearable camera facilitated this process and while issues around battery life and privacy preclude using this method in some situations, for a validity study, this provided the least intrusive method of recording the ground truth. The methods to obtain a location from the proximity function of the monitors described here are also simple to employ. Researchers looking to monitor a single location (e.g., office) or multiple locations may be able to adapt the methods we used to similar office environments. The assessment of two wear positions is a further strength. Researchers can employ a wrist-worn monitor, which may be more comfortable and less intrusive for participants, or a thigh-mounted monitor which enables the measurement of body position (sitting versus upright) [16]. Further, though direct observation (e.g., by wearable cameras) is the most accurate option for measuring location, the monitors are not only more feasible, but their limited obtrusiveness may also minimise any effect of monitoring on participant behaviour. Limitations are that these findings may not be generalisable to all environments such as those where more movement takes place and where room layouts preclude the separation of beacons (e.g. open plan offices). Additionally we collected data at 10-second intervals, therefore, the findings are also not generalizable to data collected with longer epochs. Examination of the effect of longer intervals between signal sampling and the differential effect of this longer epoch on time spent in varied levels of movement and positions are currently planned. Further research is also needed to investigate the accuracy of these devices and methods in varied work and non-work settings, with differing work patterns (e.g., longer periods of time across different locations), using different beacon layouts (for example multiple beacons in some areas) and to explore more sophisticated data treatment methods using measures of RSSI strength and movement to improve accuracy.

## Conclusions

Bluetooth proximity sensing using the ActiGraph GT9X Link monitor showed good accuracy for determining whether workers were in their office, where they spent most of their work hours and long periods of time sitting down. Accuracy was lower for locations where participants spent less time and/or spent more time moving. The accuracy of proximity detection was improved for most locations when a rule-based decision model for predicting location from multiple beacons was employed. This level of accuracy may be sufficient for researchers looking to determine where workers spend time in an office-based work environment.

## Supporting information

S1 Fig Example of received signal strength indicator output in each location for one hour for one participant. (PDF)

S2 Fig Selection of best window size in the development samples (5-fold cross-validation): highest F1-score averaged across locations. (TIF)

S1 File SAS code used to implement the multiple-locations-monitored decision model. (PDF)

S1 Table Improvement in agreement when adjusting the time-recording by -30 to +30 seconds to maximise agreement in location. (DOCX)
