# Supplementary material for: Using Bluetooth proximity sensing to determine where office workers spend time at work
Source: PLoS One. 2018 Mar 7;13(3):e0193971. doi: 10.1371/journal.pone.0193971 (PMC5841797; doi:10.1371/journal.pone.0193971)
Supplement: S1 Fig — (PDF) [file pone.0193971.s001.pdf]

RSSI values* for one participant when in various camera-determined locations (9:15-10:15 am)

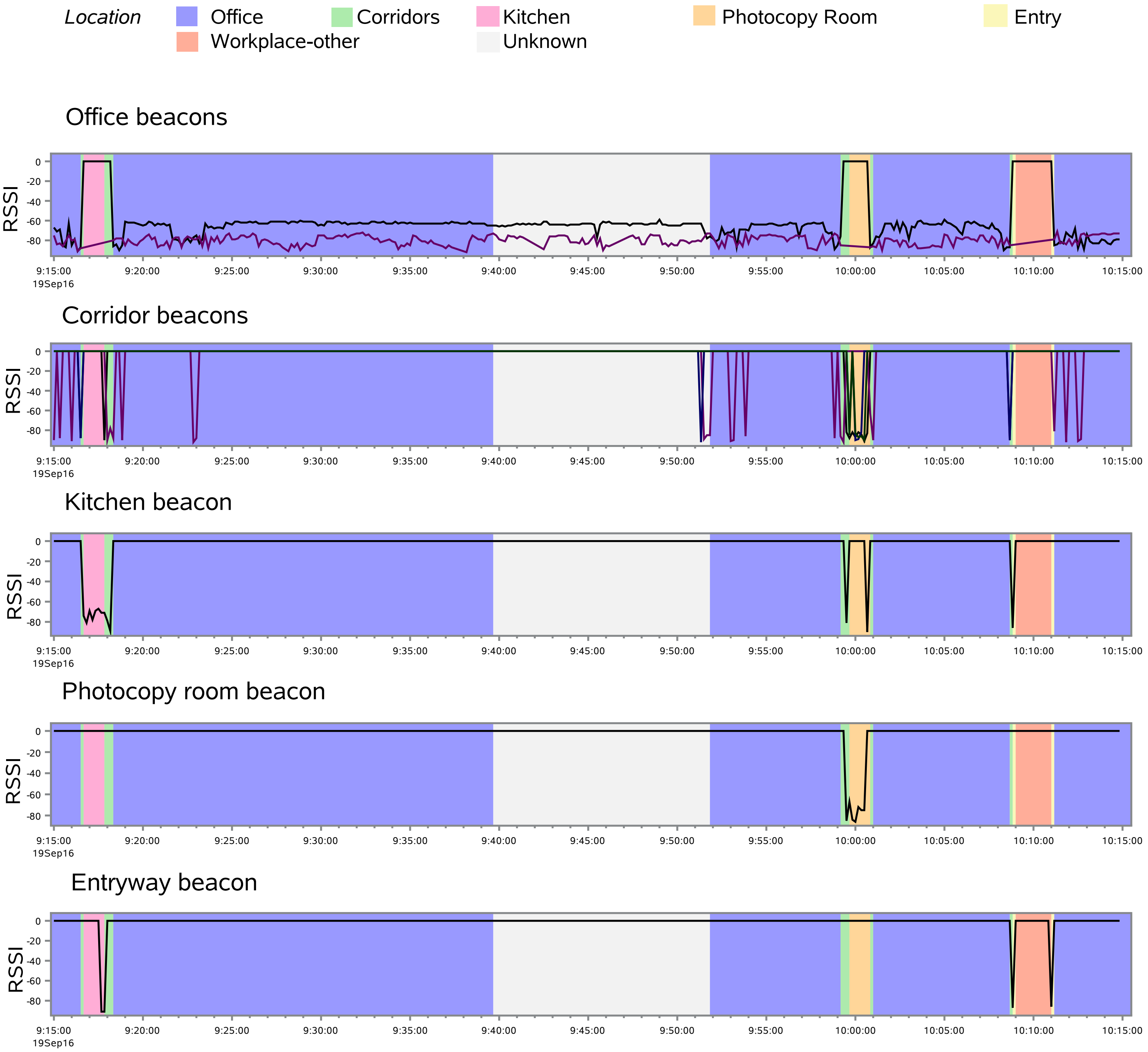

*No signal detected plotted as RSSI=0
