# Supplementary figures and images for: Using Bluetooth proximity sensing to determine where office workers spend time at work
Source: PLoS One. 2018 Mar 7;13(3):e0193971. doi: 10.1371/journal.pone.0193971 (PMC5841797; doi:10.1371/journal.pone.0193971)

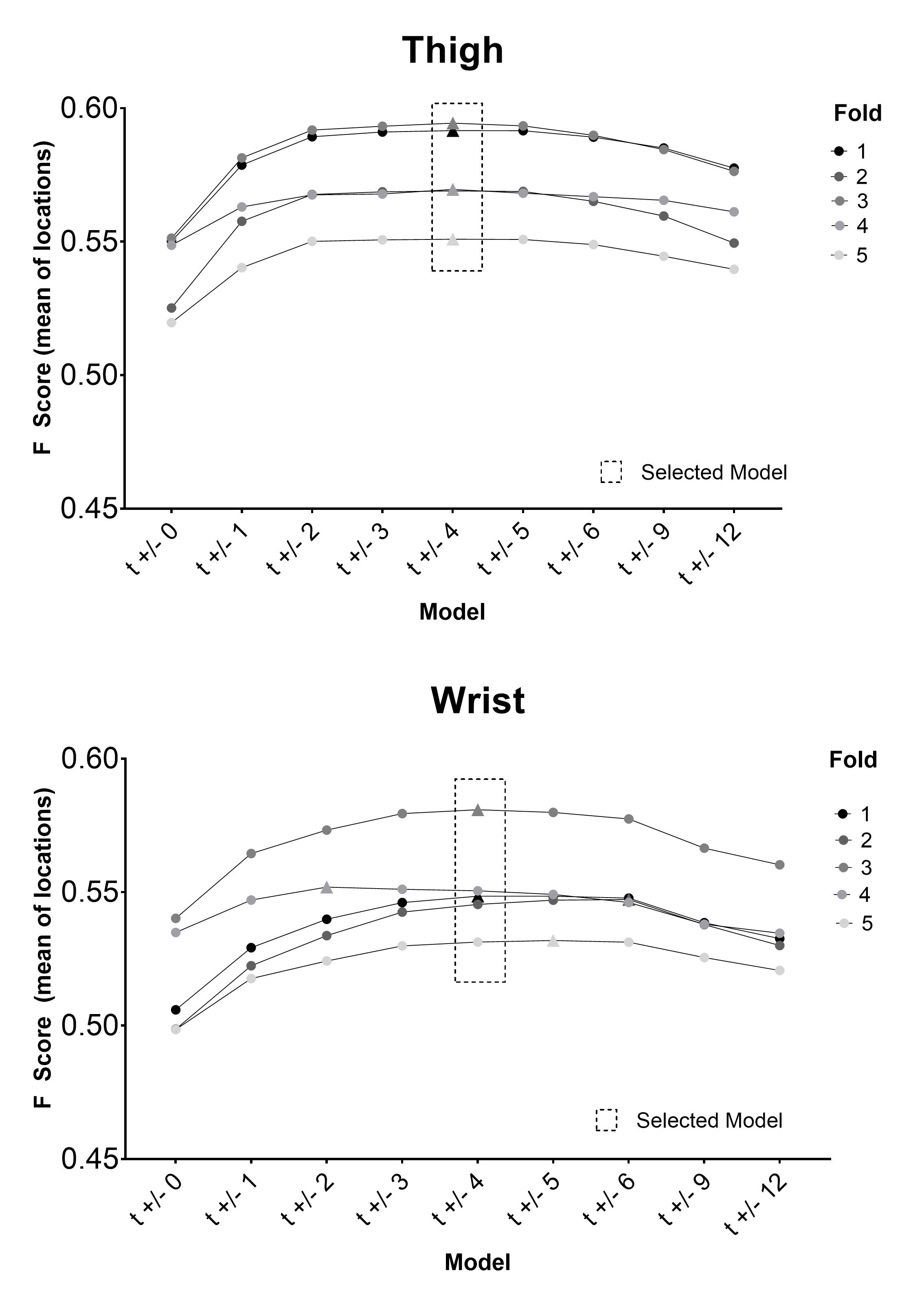

Supplement: S2 Fig — (TIF) [file pone.0193971.s002.tif]
