# Supplementary material for: Using Bluetooth proximity sensing to determine where office workers spend time at work
Source: PLoS One. 2018 Mar 7;13(3):e0193971. doi: 10.1371/journal.pone.0193971 (PMC5841797; doi:10.1371/journal.pone.0193971)
Supplement: S1 File — (PDF) [file pone.0193971.s003.pdf]

S1 File

SAS code used to implement the multiple-locations-monitored decision model.

```
********************** WHERE AND WHEN AT WORK STUDY*****************************************************
SYNTAX USED IN THE WHERE AND WHEN AT WORK STUDY TO APPLY LOCATION CLASSIFICATIONS FOR SINGLE-LOCATION-MONITORED
APPROACH AND HEURISTIC MODEL FOR MULTIPLE-LOCATIONS-MONITORED APPROACH.
INPUT DATA: ActiGraph Link files, 10-s epoch, exported as csv, with some reformatting
--csv files for each participant stored in a single folder, files begin with the participant ID (numeric), with
folder, ID and .csv being sufficient to uniquely identify files.
--Variables: 1) timestamp: date time to the nearest minute obtained from the ActiLife software
2) officedesk, officewall, Entry, Kitchen, Photocopy, wall1, wall2, wall3, and wall4 are respectively
the fields containing the Link proximity data for the sensor placed at the participant's office desk, office wall,
kitchen, photocopy room, and the 2-4 beacons [depending on the floor] placed in the corridors.
-- Row 1 after the heading contains example fake data to ensure all fields are assigned have a consistent format and
field width to suit all files and allow appending.
OUTPUT DATA: master&wearloc_&win for the specified wear location "thigh" and the specified time windows for the
majority vote used (1,3,5,7,9,11,13,19,25) with 5 being optimal in Where And When At Work, e.g., masterthigh_5
AUTHORSHIP: Elisabeth Winkler, Stewart Trost, Bronwyn Clark on behalf of the Centre of Research Excellence on
Sitting Time and Chronic Disease Prevention - Mechanisms, Measurement and Interventions
USAGE AND ADAPTATION: permitted with citation of the article in which this program appears
*****************************************************************v*********************************************;
*Label the possible location values (0-4);
proc format;
value locs_noe    1="office" 2="kitchen" 3="photocopy room" 4="corridors" 0="workplace-other";
run;
%macro link10s(id);
***** SECTION 1:
- READING IN THE DATA, ENSURING IDENTIFIERS FOR PERSON, DATE AND TIME ARE PRESENT AND SUFFICIENTLY DETAILED
- FOR EACH EPOCH, DETERMINING SIGNAL PRESENCE/ABSENCE AT EACH BEACON AND PARTICIPANT PRESENCE/ABSENCE AT EACH
LOCATION BY THE ONE-LOCATION MONITORED APPROACH
- FOR EACH EPOCH, DETERMINING AT HOW MANY LOCATIONS ONE OR MORE BEACON HAD REGISTERED A SIGNAL AS PRESENT
SIMULTANEOUSLY *****;
%let rest = *;
*** files began with the participant ID;
PROC IMPORT OUT= WORK.p_&id DATAFILE= "&dir&id&rest..csv"  DBMS=CSV REPLACE;
     GETNAMES=YES; DATAROW=2;
RUN;
```

```
data p_&id; set p_&id;
if _N_=1 then delete; *** deleting _N_ = 1 –not real data; if timestamp = . then delete; **no data;
PID = &id; *Create a varaible for the participant ID;
array link officedesk officewall Entry   Kitchen Photocopy wall1 wall2 wall3 wall4;
do over link; if link ~=. then link =1; if link =. then link =0; end; *reduce to sensor signal present/not;
*single-location-monitored approach: office_any, walls_any, kitchen, photocopy, entry;
office_any =max(of officewall, officedesk); *signal present in the office [desk or wall beacons]?;
walls_any = max(of wall1, wall2, wall3, wall4); *signal present in the corridors [at any of the wall beacons]?;
keep Timestamp pid officedesk officewall wall1 wall2 wall3 wall4 Entry   Kitchen   Photocopy office_any walls_any;
run;

*calculate which which 10-second epoch applies to each record within each timestamp;
data p_&id; set p_&id; by pid timestamp; if first.timestamp then do;  epn=1;  end; epn+1; run;
data maxep; set p_&id; by pid timestamp; if last.timestamp; lastepn=epn; keep pid timestamp lastepn; run;
data p_&id; merge p_&id maxep; by pid timestamp; sec = 10*(epn - lastepn + 5);  drop epn lastepn; run;
proc datasets nolist; delete maxep; run; quit;

***** SECTION 2: The majvote majority voting macro that
- OBTAININS INFORMATION REQUIRED FOR HEURISTIC MODEL [A,B]:
--for each timewindow, calculating A1) the number of times each beacon registered a signal and A2) the maximum
number of times a beacon registered a signal
--for each epoch, calculating B) the number of locations registering a signal simultaneously
- APPLYIS THE HEURISTIC MODEL TO CLASSIFY LOCATION [C] *****;
%macro majvote(win);
proc expand data = p_&id out=pnew&wearloc._&win._&id ; *A1;
convert officewall = sum_officewall/ method = none transformout = (cmovsum &win);
convert officedesk = sum_officedesk/ method = none transformout = (cmovsum &win);
convert kitchen = sum_kitchen/ method = none transformout = (cmovsum &win);
convert photocopy = sum_photocopy/ method = none transformout = (cmovsum &win);
convert wall1= sum_wall1/ method = none transformout = (cmovsum &win);
convert wall2= sum_wall2/ method = none transformout = (cmovsum &win);
convert wall3= sum_wall3/ method = none transformout = (cmovsum &win);
convert wall4= sum_wall4/ method = none transformout = (cmovsum &win);
run;

data pnew&wearloc._&win._&id ;set pnew&wearloc._&win._&id ;
maxval = max(of sum_officewall, sum_officedesk, sum_kitchen, sum_photocopy, sum_wall1, sum_wall2, sum_wall3,
sum_wall4);  *A2;
sum_locs = office_any + kitchen + photocopy + walls_any; *B;
```

```
*HEURISTIC MODEL: C1) If 0 locations recorded a signal: -> location = workplace-other;
if sum_locs = 0 then do; linkpos = 0; *workplace-other; end;
*HEURISTIC MODEL: C2)  If 1 locations recorded a signal: -> location = location with the signal;
if sum_locs = 1 then do; if photocopy=1 then linkpos = 3; if kitchen=1 then linkpos = 2; if walls_any=1 then linkpos
= 4; if office_any=1 then linkpos = 1; end;
*HEURISTIC MODEL: C3) If >1 locations registered signals simultaneously location -> location with the greatest
number of signals recorded as present during the
timewindow [majority vote], resolving "ties" for most common by prevalence ranking;
* each subsequent classification overwrites the previous: put lowest prevalence location first and highest
prevalence location last;
if sum_locs >1 then do;
if sum_photocopy=maxval then linkpos = 3;*photocopy room the 4th most prevalent;
if sum_kitchen=maxval then linkpos = 2;*kitchen, 3rd most prevalent;
if sum_walls=maxval then linkpos = 4;*corridors, 2nd most prevalent;
if sum_officewall=maxval then linkpos = 1;*office wall beacon, office = 1st most prevalent;
if sum_officedesk=maxval then linkpos = 1;*office desk beacon, office = 1st most prevalent;
if maxval = 0 then linkpos = 0; *no sensor recorded;
end;
format linkpos locs_noe.;
run;
%if &run = 1 %then %do; data master&wearloc._&win; set pnew&wearloc._&win._&id; run; %end; *CREATE new master
dataset from data of first participant;
%if &run = 2 %then %do; data master&wearloc._&win; set master&wearloc._&win pnew&wearloc._&win._&id; run;
%end;*APPPEND data from additional participants to the master dataset;

proc datasets nolist; delete pnew&wearloc._&win._&id; run; quit;
%mend majvote;
%majvote(1);  %majvote(3);  %majvote(5);  %majvote(7);  %majvote(9);  %majvote(11); %majvote(13); %majvote(19);
%majvote(25);*execute majvote macro for the nominated timewindows;
proc datasets nolist; delete  p_&id; run; quit;
%mend link10s;

%let dir=C:\Documents\ProjectWWaW\LinkData\Thigh\; *indicate folder that contains the relevant csv files;
%let wearloc = thigh; *indicate the wear location - here, thigh;
*execute the macro for all participants, specifying run to be 1 for the first participant and 2 thereafter for
subsequent participants;
%let run = 1;
%link10s(01);
%let run = 2; *subsequent participants-> append to existing master dataset;
```

```
%link10s(02);
%link10s(03);
```
