# Supplementary material for: Using Bluetooth proximity sensing to determine where office workers spend time at work
Source: PLoS One. 2018 Mar 7;13(3):e0193971. doi: 10.1371/journal.pone.0193971 (PMC5841797; doi:10.1371/journal.pone.0193971)
Supplement: S1 Table — (DOCX) [file pone.0193971.s004.docx]

**S1 Table**: Improvement in agreement when adjusting the time-recording by -30 to +30 seconds to maximise agreement in location (percentage correct).

|  | **Thigh** | **Wrist** |
| --- | --- | --- |
| **n individuals** | 30 | 30 |
| **n observations** ^a^ | 55266 | 55266 |
| **n camera files** | 181 | 126 |
| **Alignment with maximum agreement** |  |  |
| Camera 30s ahead of monitor | 0 (0%) | 0 (0%) |
| Camera 20s ahead of monitor | 0 (0%) | 0 (0%) |
| Camera 10s ahead of monitor | 0 (0%) | 0 (0%) |
| Same time | 143 (79.0%) | 125 (69.1%) |
| Camera 10s behind monitor | 17 (9.4%) | 13 (7.2%) |
| Camera 20s behind monitor | 8 (4.4%) | 27 (14.9%) |
| Camera 30s behind monitor | 13 (7.2%) | 16 (8.8%) |
| **Improvement in F scores (aligned - unaligned)** |  |  |
| Office | 0.000 | 0.000 |
| Kitchen | -0.005 | -0.002 |
| Photocopy Room | -0.005 | 0.006 |
| Corridors | -0.064 | -0.039 |
| Workplace - other | -0.011 | -0.008 |

^a^ Limited to the observations that can be time-matched between the monitor and video camera with all possible time shifts (-30 to +30 seconds).
